# Effects of pre-oxidation temperature and air volume on oxidation thermogravimetric and functional group change of lignite

**DOI:** 10.1371/journal.pone.0316705

**Published:** 2025-01-07

**Authors:** Baoshan Jia, Zihao Chai, Wanting Zhao, Xian Wu

**Affiliations:** 1 College of Safety Science and Engineering, Liaoning Technical University, Fuxin, Liaoning, China; 2 Key Laboratory of Mine Thermodynamic Disaster & Control of Ministry of Education, Liaoning Technical University, Huludao, Liaoning, China; University of Sharjah, UNITED ARAB EMIRATES

## Abstract

To investigate the impact of the oxidation temperature and variations in airflow conditions on coal spontaneous combustion characteristics, pre-oxidized coal samples were prepared using a programmed temperature rise method. Synchronous thermal analysis experiments and Fourier transform infrared spectroscopy were conducted to explore changes in the thermal effects and functional group content of the coal samples, respectively. The results indicate that variations in pre-oxidation conditions primarily in fluence the activation temperature and maximum weight loss temperature of the coal samples, while exerting a lesser impact on the critical temperature and ignition point. Variations in air volume conditions predominantly affect the content of Ar-C-O- and -CH_2_ & -CH_3_ in the oxygen-containing functional group region. The trend of the average activation energy within a conversion rate range of 0.2 to 0.6 of pre-oxidized coal samples changing with the increased of pre-oxidation temperature under the air flow conditions of 25mL/min and 50mL/min is consistent, but opposite to that under the air flow conditions of 100mL/min and 200mL/min. Compared to raw coal, under an airflow rate of 50 mL/min and when oxidized to 110°C, the coal sample exhibits an increase in the content of OH…OH, accompanied by reductions in the critical temperature, activation temperature, ignition point, and maximum weight loss temperature to varying degrees, thereby rendering it more susceptible to oxidative spontaneous combustion.

## 1. Introduction

Coal serves as the cornerstone of China’s energy security and is a crucial fossil fuel in the fields of power engineering, metallurgy, energy chemicals, and others [1, 2]. Spontaneous combustion of coal often occurs in the process of coal mining and utilization. Coal spontaneous combustion not only affects the utilization of coal resources but also indirectly leads to secondary disasters such as gas and dust explosions, posing a threat to personnel safety and environmental health [3, 4]. During the primary oxidation process, coal may be influenced by various factors or measures such as cooling and oxygen deficiency, leading to its temperature cooling down to normal temperature slowly before reaching the ignition point, which is referred to as pre-oxidized coal [5–7]. The peak temperature achieved during the primary oxidation process of coal is referred to as the pre-oxidation temperature, which is typically closely correlated with the degree of oxidation of the coal [8–10]. High airflow volume and velocity can enhance the kinetic energy of air penetration into the coal mass, ensuring continuous oxygen supply. However, excessively high airflow may also dissipate the heat generated by surface oxidation in the coal pile, thereby influencing the occurrence of spontaneous combustion [11]. The goaf’s air leakage intensity fluctuates due to coal mine production activities, and the spontaneous combustion of coal becomes more complex due to uncertainties surrounding air volume and other oxidation conditions [12, 13]. Therefore, it is of great significance to study the change of conditions in the pre-oxidation process and the characteristics of coal spontaneous combustion process for preventing and controlling the occurrence and development of coal spontaneous combustion disasters.

Thermal effects not only influence the severity of coal spontaneous combustion disaster consequences but also constitute a significant factor affecting the rate of coal oxidation processes [14–16]. Pan et al. [17] utilized a C600 microcalorimeter to analyse the heat release variation of raw coal and water-soaked coal under air volume of 25, 50, 75, and 100 mL/min. Li et al. [18] investigated the stages of coal spontaneous combustion and indicative gases using thermogravimetric analysis, dividing the temperature rise process during coal combustion into the latency period, the slow self-heating period, the accelerated self-heating period, and the combustion period. Li et al. [19] conducted a study on the thermal behavior of coal under varying oxygen concentrations and heating rates using the synchronous thermal analysis method. Xiao et al. [20] investigated the effect of air volume of 90, 120, 150, and 180 mL/min on the spontaneous combustion of bituminous coal using the TG-DSC method. The study revealed that the characteristic temperature was the lowest and the reactivity between coal and oxygen was the highest at an airflow rate of 150 mL/min. Li et al. [21] studied the influence of air volume on the combustion kinetics of non-caking coal through thermogravimetric analysis. The results indicated that as the ventilation rate increased, the processes of oxygen adsorption, thermolysis, and combustion of the coal advanced, leading to shortened durations of water moisture evaporation and gas desorption, while prolonging the oxygen adsorption and combustion processes. Zhao et al. [22] utilized simultaneous thermoanalysis to compare the exothermic characteristics of fresh coal and weathered coal under four heating rates, identifying 270°C as the key temperature for spontaneous combustion in weathered coal. Synchronous thermal analysis test is commonly utilized to investigate the exothermic characteristics of samples and can characterize the spontaneous combustion properties of coal by analyzing the characteristic temperature points on the curves.

Differences in the oxidation process are often related to variations in both intrinsic coal properties and external environmental conditions. The lower the degree of coal metamorphosis, the more susceptible it is to oxidation and spontaneous combustion, meaning that under identical conditions, lignite exhibits a higher propensity for spontaneous combustion compared to bituminous coal [23–25]. Nie et al. [26] investigated the spontaneous combustion and oxidation characteristics of coals with varying degrees of metamorphism. The finding suggested that with the intensification of coal metamorphism, there is a corresponding increase in the three distinct temperature points (critical temperature, drying temperature, and activation temperature). In order to explore the characteristics of secondary oxidation or reignition of coal, some scholars have studied the differences in its pre-oxidation process. Wang et al. [27] utilized the temperature-programmed oxidation technique to generate oxidized coal samples at predetermined temperatures of 50, 70, 90, 110, and 130°C. It was found that the pre-oxidized coal sample at 130°C exhibited a higher propensity for spontaneous combustion compared to the raw coal. Xiao et al. [28] prepared pre-oxidized coal samples at preset temperatures of 90, 150, and 200°C using a programmed temperature rise method and examined the thermophysical properties of the coal samples using the laser flash technique. The study found that pre-oxidation altered the aromatic microcrystalline structure and pore structure within the coal samples, thereby affecting heat transfer in the coal matrix. Xu et al. [29] employed simultaneous thermoanalytical methods to investigate the influence of the coupling effect of stress and pre-oxidation temperature on the macroscopic characteristic temperatures of coal spontaneous combustion. Variations in pre-oxidation temperature indirectly indicate different levels of oxidation achieved by coal during its initial oxidation process, thereby influencing the progression of sustained or recurrent oxidation in coal.

In summary, there have been studies on the effect of oxidation conditions on coal oxidation spontaneous combustion, but there are relatively few studies on the coupling of pre-oxidation temperature and air volume. To better simulate the oxidation process of coal samples in actual gob areas, this article utilized a programmed temperature rise method to prepare pre-oxidized coal samples under different preset temperatures and airflow conditions. Subsequently, Fourier Transform Infrared spectroscopy was employed to measure changes in the functional group content of the coal samples. Additionally, synchronous thermoanalysis method was utilized to analyze the differences in characteristic temperatures and heat release intensities between raw coal and pre-oxidized coal. Finally, the apparent activation energy was calculated using the Kissinger–Akahira–Sunose (KAS) method to indirectly characterize the risk of coal spontaneous combustion. It provides theoretical guidance for further understanding the influence of different oxidation conditions on coal spontaneous combustion characteristics and preventing coal spontaneous combustion during coal mining, storage and transportation.

## 2. Materials and methods

### 2.1 Coal samples

The coal samples used in this study are from the Zuoyun Donggucheng Coal Mine of the Coal Import and Export Group Company in Shanxi, China. The contents of moisture, ash, volatile, and fixed carbon in the lignite are 12.18%,14.07%,34.30%, and 39.45%, respectively. The fresh coal was crushed with a hydraulic crusher under the vertical pressure of 25MPa, and sieved to obtain the coal sample with a particle size below 10mm for backup.

The highest temperature of coal sample in the pretreatment process is the target temperature set by the experiment, that is, the pre-oxidation temperature conditions (40°C, 70°C, 110°C, 140°C, 170°C). The programmed heating experiment was conducted to oxidize 200g of coal samples in an air atmosphere at a heating rate of 0.5°C/min, under four different air volume conditions (25 mL/min, 50 mL/min, 100 mL/min, 200 mL/min),respectively, within a temperature range from room temperature to specific target temperatures. After the coal samples were cooled to room temperature in a nitrogen environment, pre-oxidized coal samples were obtained and appropriately labeled. The superscript of R denotes the preset airflow condition, while the subscript represents the preset final oxidation temperature. Raw coal is denoted as R_0_.

### 2.2 Fourier transform infrared spectroscopy test

Spectral characteristics of coal samples were measured using the Nicolet iS50 Fourier transform infrared spectrometer developed by Thermo NICOLET Corporation, USA. In order to avoid the influence of moisture on infrared spectrum absorption, the coal sample was dried in a vacuum drying oven at 105°C. The coal sample, after drying and sieved to below 200 mesh(<0.075 mm), was mixed with the alkali metal halide (KBr) at a ratio of 1:180. After grinding evenly in an agate mortar, 0.2g mixture is put into a tablet press to produce ingots. Infrared wavenumber measurement range is 400 cm^−1^–4000 cm^−1^, resolution is 4 cm^−1^, a total of 64 scans, each group of samples repeated test twice.

### 2.3 Synchronous thermal analysis experiment

The Synchronous thermal analyzer STA449C (Netzsch) was utilized to ascertain the characteristic parameters and variations in heat flow for both raw coal and pre-oxidized coal samples. Prior to conducting synchronized thermal analysis experiments on coal samples with particle sizes below 0.125 mm, the ceramic crucible was preheated for 15 minutes to mitigate the interference of moisture, thereby maximizing the accuracy of the experimental data. Approximately 10 mg of coal sample was placed in a crucible for each experiment, with heating rates of 10°C/min, 20°C/min, and 30°C/min, respectively. The coal samples were subjected to heating from ambient temperature to 700°C under an experimental atmosphere comprising of air volume of 100 mL/min, containing 21% oxygen.

### 2.4 Calculation method of activation energy

The spontaneous combustion of coal is a burning phenomenon induced by an oxidation reaction. The kinetic analysis method of thermal effects can be employed to calculate the minimum energy needed for the oxidation reaction, known as the activation energy [30]. The basic kinetic equation is as follows [31, 32].

$$\frac{d\alpha }{dT}=\frac{A}{\beta }\text{exp}(-\frac{Ea}{RT})f(\alpha )$$ (1)

$$\alpha =\frac{m_{0}-m_{1}}{m_{0}-m_{2}}$$ (2)

Where *α* represents the mass loss rate of the sample; *β* is the constant heating rate, (K/min); Ea is the apparent activation energy, (kJ/mol); *m*_0_ represents the initial coal mass,(g); *m*_1_ represents the coal mass at the time of t,(g); *m*_2_ represents the coal mass at the end of the test,(g).

The equal conversion rate method simplifies the process of solving the activation energy, and does not require the determination of the mechanism function of the reaction, and the reaction rate is only related to the temperature, so the calculated activation energy is more accurate. The typical Kissinger–Akahira–Sunose integral method within the equal conversion rate approach is expressed as follows [33, 34].

$$Ln(\frac{\beta }{T^{2}})=Ln(\frac{AR}{E_{\alpha }g(\alpha )})-\frac{Ea}{RT}$$ (3)

Where *A* is the pre-exponential factor; *R* is the molar gas constant, (8.314J/[mol∙k]); *T* is the coal sample temperature (k).

## 3. Results and discussion

### 3.1 Rule of functional group change

**Fig 1:**
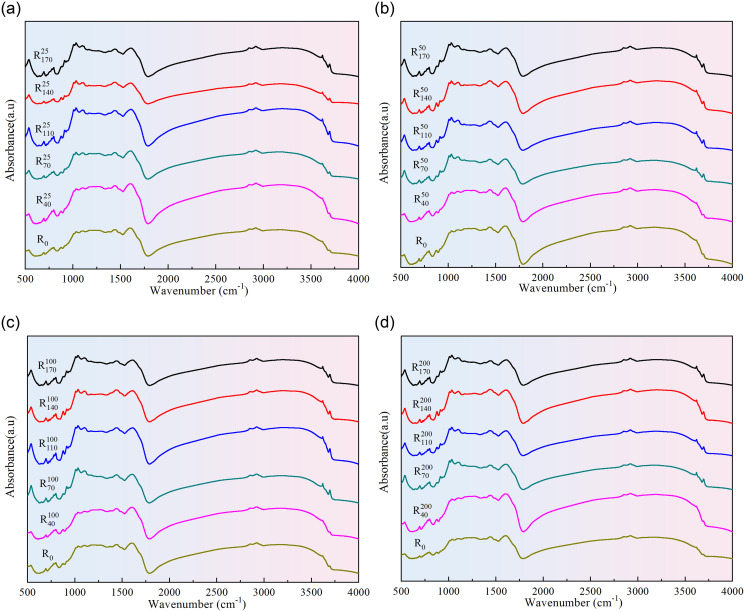
Original FTIR diagram of raw coal and pre-treated coal samples.

Fourier transform infrared spectroscopy method can be used to determine the functional groups on the surface of coal samples according to different group vibration frequencies. The FTIR spectra of raw coal and pre-oxidized coal samples are illustrated in Fig 1. Based on the positions of infrared characteristic absorption peaks and relevant literature, the spectral curve can be divided into three regions for analysis: the oxygen-containing functional group region at 1000–1800 cm^−1^, the aliphatic region at 2800–3000 cm^−1^, and the hydroxyl structural region at 3000–3750 cm^−1^ [35–37]. As can be seen from Fig 1, the position of characteristic peaks in FTIR spectra does not shift significantly with the change of coal sample pre-oxidation conditions, while the sharpness of characteristic peaks in the functional group structure area fluctuates significantly.

**Fig 2:**
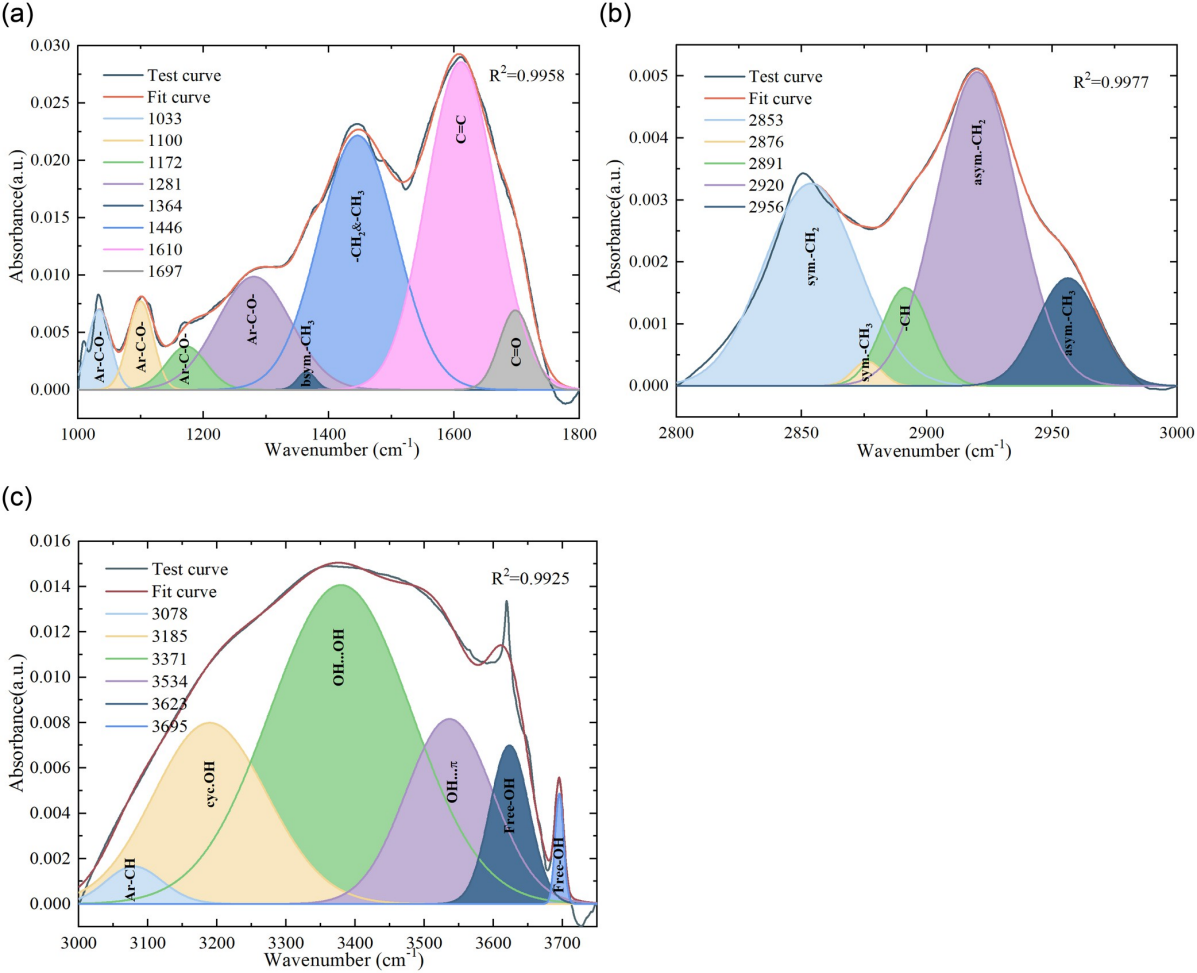
Fitting process of Fourier infrared spectrum: (a) Wave numbers range from 1000 to 1800cm^-1^, (b) Wave numbers range from 2800 to 3000cm^-1^ and (c)Wave numbers range from 3000 to 3750cm^-1^.

Absorbance is the negative logarithm of light transmittance, usually indicating the intensity of the sample’s absorption of infrared light. For semi-quantitative analysis of the functional groups on the coal surface, peak separation of the spectral curves was conducted using Gaussian fitting in PeakFit V4.2 software. The confidence coefficients of all fitted curves were greater than 0.99. Taking the FTIR spectral fitting of $R_{40}^{25}$ as an example, the peak-separated fitting results for the three wavenumber intervals are shown in Fig 2.

#### 3.1.1 Content analysis of oxygen-containing functional groups region

**Fig 3:**
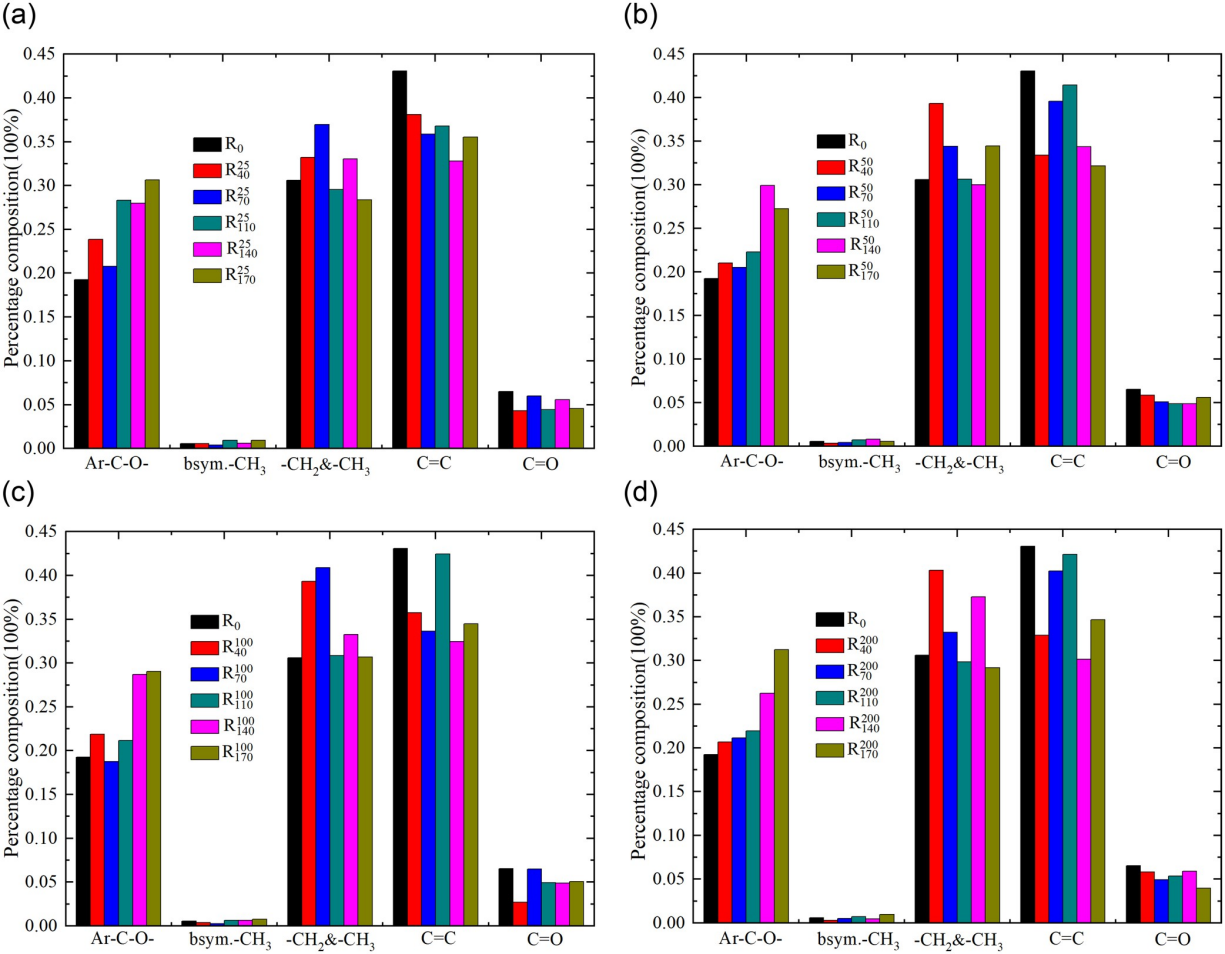
Distribution of functional groups of raw coal and pre-oxidized coal samples in 1000-1800cm^-1^ band.

The total peak area represents the summation of individual peak areas within the specified wavenumber range. The content distribution of functional group is represented by the ratio of the peak area of that functional group to the total peak area. As illustrated in Fig 3, except for a 2.62% decreased in the content of phenolic, alcoholic, ether, and ester oxygen bonds (Ar-C-O-) in the coal sample oxidized to 70°C under an airflow rate of 100 mL/min compared to the raw coal, the content of Ar-C-O- in coal samples under other pre-oxidation conditions approximately increased by 0.06–0.62 times relative to the raw coal. Meanwhile, the content of carbonyl group (C = O) in the pre-oxidized coal samples decreased to varying degrees compared to the raw coal. A higher content of Ar-C-O indicated stronger oxidative activity in coal samples, while the lower content of C = O in pre-oxidized coal samples compared to raw coal may be associated with oxidative decomposition during the pretreatment process. In comparison with coal samples subjected to various pre-oxidation conditions, the coal sample oxidized to 70°C under an airflow rate of 100 mL/min exhibited the lowest concentrations of Ar-C-O- and methyl groups in symmetrical bending vibration mode (bsym.-CH_3_). Conversely, it demonstrated the highest concentrations of methylene and methyl groups in asymmetrical bending vibration mode (-CH_2_ & -CH_3_) and C = O. This suggest a relatively lower oxidative activity for this sample, implying that a larger energy barrier may needs to be overcome for reoxidation to occur.

Under identical pre-oxidation airflow conditions, the content of -CH_2_ & -CH_3_ in coal samples exhibited an overall approximate decreasing trend as the oxidation temperature increased. Conversely, the content of Ar-C-O- displayed an overall increasing trend with the elevation of oxidation temperature. The overall fluctuation trend of other functional groups within the oxygen-containing functional group region was relatively small as the oxidation temperature increases. It showed that the pre-oxidation temperature conditions will have different degrees of influence on the content of functional groups in the preoxidized coal samples.

Under identical oxidation temperature conditions, the trend in the variation of the -CH_2_ & -CH_3_ content in coal samples with increasing pre-oxidation airflow rate was opposite to that of bsym.-CH_3_ content. However, the overall fluctuation trend of bsym.-CH_3_ content was relatively small. For coal samples oxidized to 40°C, the content of -CH_2_ & -CH_3_ showed an overall upward trend as the air volume increases, and the content of -CH_2_ & -CH_3_ increased by 0.18 times when the air volume increased from 25mL/min to 50mL/min. However, for coal samples oxidized to 70°C, the content of -CH_2_ & -CH_3_ exhibited an overall approximate downward trend as the airflow condition increased. This indicated that variations in pre-oxidation airflow conditions can also impact the content of functional groups in coal samples under the same oxidation temperature condition. As the pre-oxidation airflow rate increased, the overall Ar-C-O- content in coal samples oxidized to 110°C exhibited an decreasing trend, with a significant reduction of 21.30% when the airflow rate increased from 25 mL/min to 50 mL/min. For coal samples oxidized to 140°C and 170°C, the fluctuation trends of Ar-C-O- content and -CH_2_ & -CH_3_ content were relatively significant as the airflow conditions increased. The supply of air volume facilitates effective contact between coal and oxygen on one hand, yet hinders the accumulation of coal heat on the other. Variations in airflow conditions primarily influence the content of Ar-C-O- and -CH_2_ & -CH_3_ in the oxygen-containing functional group region.

#### 3.1.2 Analysis of aliphatic regional content

**Fig 4:**
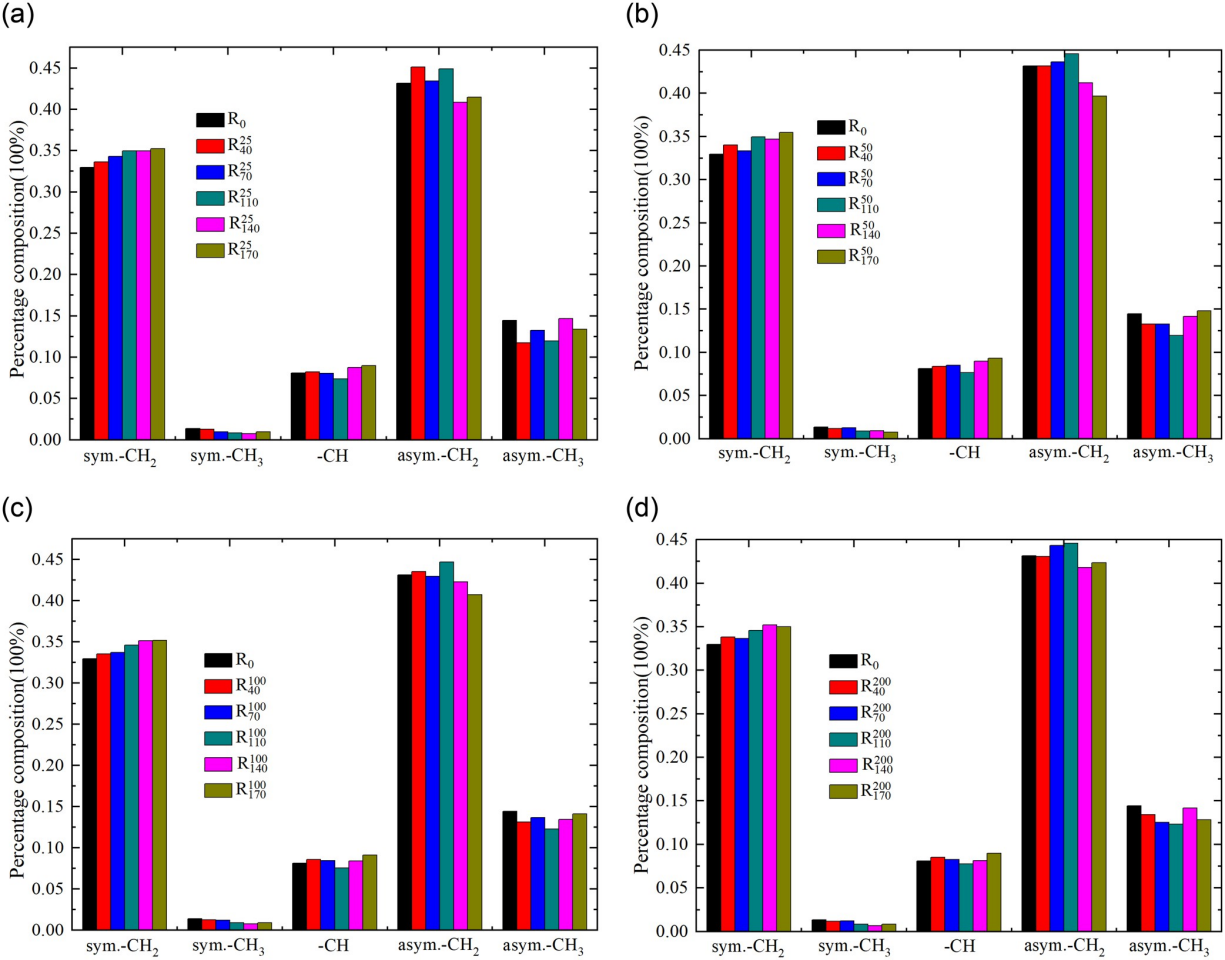
Distribution of functional groups of raw coal and pre-oxidized coal samples in 2800-3000cm^-1^ band.

The coal samples in the aliphatic region mainly include two forms of methylene and methyl stretching vibration, as well as naphthenes or aliphatic C-H bond stretching vibration. As shown in Fig 4, the content of methylene (sym.-CH_2_) in the form of symmetric stretching vibration in coal samples under different preoxidation conditions was approximately 0.01 to 0.07 times higher than that of raw coal, while the content of methyl (sym.-CH_3_) in the form of symmetric stretching vibration was approximately 4.72% to 49.29% lower than that of raw coal. During the pre-oxidation process, the coal sample likely consumes a substantial amount of sym.-CH_3_, and undergoes oxidation to produce a lesser sym.-CH_2_. Compared to coal samples under different pre-oxidation conditions, the coal sample oxidized to 40°C with an air flow rate of 25 mL/min exhibited the highest content of methylene groups in asymmetric stretching vibration (asym.-CH_2_), which was 0.04 times higher than that of the raw coal. Conversely, it displayed the lowest content of methyl groups in asymmetric stretching vibration (asym.-CH_3_), representing a 18.80% decrease compared to the raw coal. This shows that long-chain aliphatic hydrocarbons are decomposed in coal samples oxidized to 40°C at 25 mL/min air flow rate, and coal sample may have higher reactivity and are more inclined to react with oxygen.

Under identical pre-oxidation airflow conditions, the trends in the content of asym.-CH_3_ and asym.-CH_2_ in coal samples exhibited opposite changes as the oxidation temperature increased, with a relatively more significant decreasing trend observed in the content of asym.-CH_2_. The trends in the content changed of asym.-CH_3_ and asym.-CH_2_ in pre-oxidized coal samples, with respect to increasing oxidation temperature, were relatively more pronounced at airflow conditions of 25 mL/min and 50 mL/min compared to those at 100 mL/min and 200 mL/min. However, the fluctuation in the functional group content within the aliphatic region of pre-oxidized coal samples, in response to variations in oxidation temperature and air volume conditions, is relatively minor.

#### 3.1.3 Hydroxyl structure region content analysis

**Fig 5:**
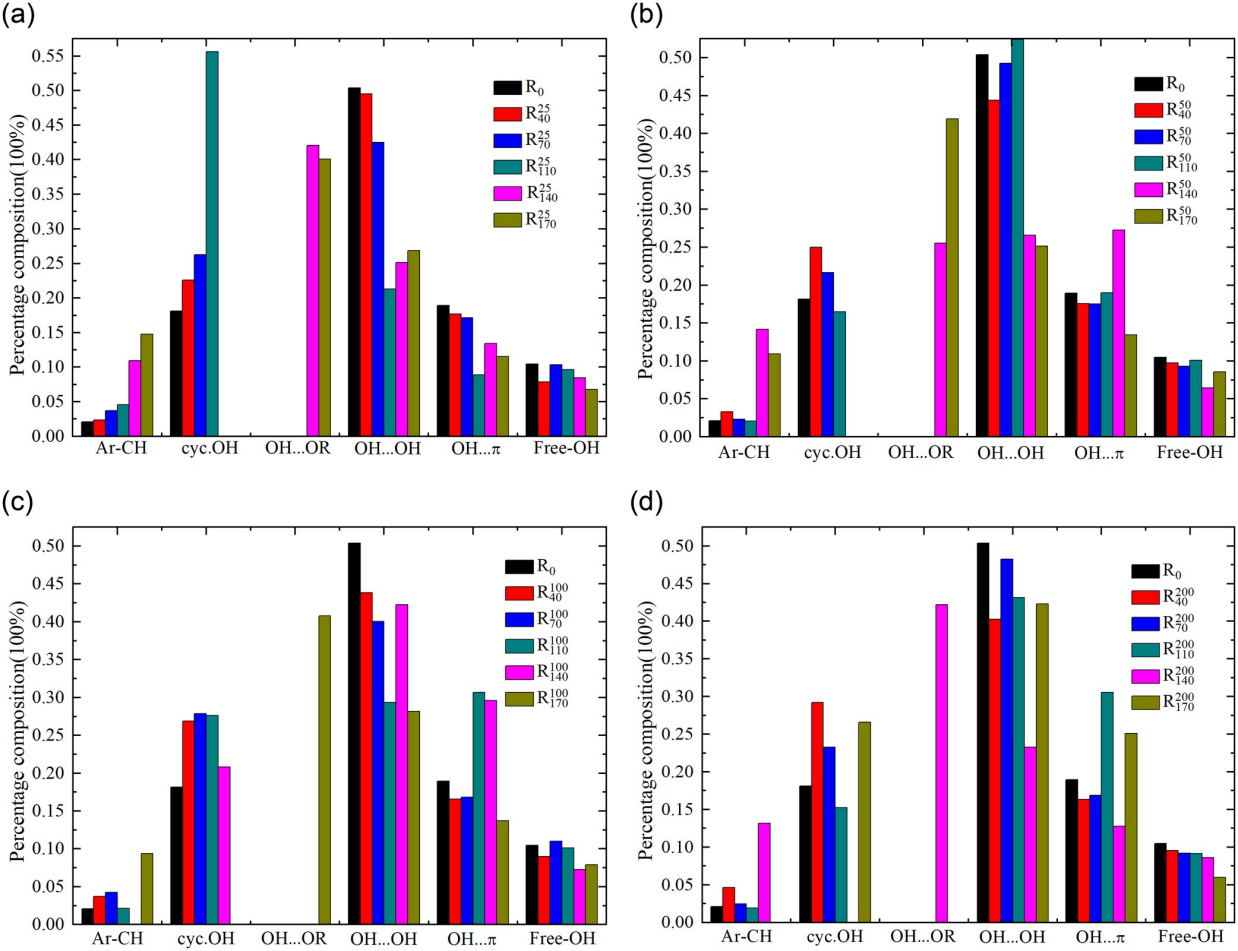
Distribution of functional groups of raw coal and pre-oxidized coal samples in 3000-3750cm^-1^ band.

Hydroxyl group is an important active group and intermediate product in coal-oxygen reaction [38]. As illustrated in Fig 5, except for the condition of 100 mL/min airflow where the coal sample oxidized to 70°C exhibited an increase in the free hydroxyl group (Free-OH) content by a factor of 0.05 compared to the raw coal, the Free-OH content in coal samples under other pre-oxidation conditions decreased approximately by 0.85% to 42.62% relative to the raw coal. It indicates that variations in oxidation conditions affect the oxidative decomposition of hydroxyl groups in a free state within coal samples during the pretreatment process. Except for the coal sample oxidized to 110°C under an air flow rate of 50 mL/min, which exhibited an increase in self-association hydroxy-hydrogen bond (OH…OH) content by a factor of 0.04 compared to the raw coal, the OH…OH content of coal samples under other pre-oxidation conditions decreased approximately by 1.68% to 57.73% relative to the raw coal. This may be related to varying degrees of consumption of phenolic and alcoholic hydroxyl groups existing in intramolecular association forms under different oxidation conditions.

Under identical pre-oxidation air flow conditions, the Free-OH content in coal samples exhibited an overall decreasing trend with an increase in oxidation temperature, accompanied by relatively minor fluctuations. The contents of OH…OH, the hydroxy π hydrogen bond (OH…π) and the hydroxyl ring hydrogen bond (cyc.OH) in the pre-oxidized coal sample fluctuated greatly before and after 110°C with the increase of oxidation temperature, which may be related to the structural change of the coal sample. Under the condition of 25mL/min preoxidation air volume, the content of aromatic hydrocarbon carbon-hydrogen bond (Ar-CH) in the pre-oxidized coal sample continued to increase as the preset temperature rised between 40 and 170°C, which favored the enhancement of the oxidation activity of the coal sample. Under pre-oxidation airflow conditions of 50 mL/min, 100 mL/min, and 200 mL/min, the content of Ar-CH in the pre-oxidized coal samples exhibited an approximate decreasing trend prior to 110°C as the oxidation temperature condition increased. However, after reaching 110°C, an increasing trend in Ar-CH content was observed.

Under the same oxidation temperature condition, the content of OH…OH and cyc.OH of coal samples had a relatively large trend of change with the increased of air volume, while the content of Free-OH had a relatively small fluctuation with the increased of air volume. The contents of Ar-CH and cyc.OH in coal samples oxidized to 40°C increased with the augmentation of pre-oxidation airflow conditions, whereas the trends in OH…π and OH…OH contents exhibited an opposite pattern, significantly influencing the oxidation characteristics. For coal samples oxidized to 70°C and 110°C, the trend of OH…OH content change with the increase of pre-oxidized air volume conditions was always opposite to that of cyc.OH content. However, when the oxidation temperature condition was 140°C, the content of OH…OH and OH…π in the coal sample showed a upward trend with the increase of air volume before 100mL/min, while the opposite trend occurs after 100mL/min. The contents of OH…OH and OH…π in coal samples oxidized to 170°C exhibited an approximately increasing trend with the elevation of pre-oxidation airflow conditions. It shows that the content of functional groups in coal samples with different oxidation degree is affected by the change of air volume. The changes of OH…OH, OH…π and cyc.OH content may be closely related.

### 3.2 Changes in heat release characteristics

**Fig 6:**
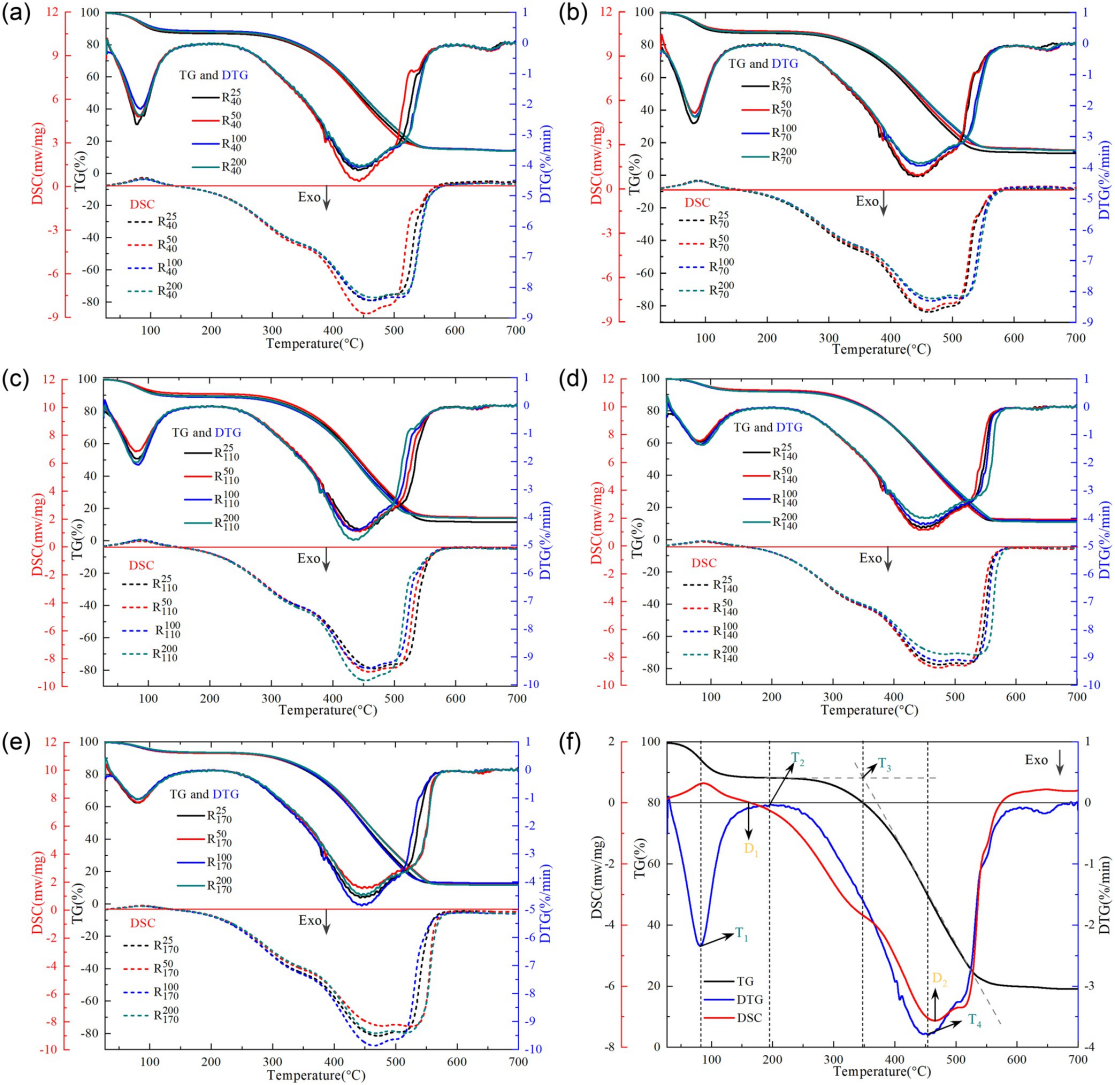
Thermal analysis curves of raw coal and pre-oxidized coal samples.

As shown in Fig 6, under the same oxidation temperature condition, the change of pre-oxidation air volume condition has a great influence on the extreme value of mass loss rate and heat release intensity on the derivative thermogravimetry (DTG) curve of coal sample. The higher rate of mass loss and exothermic intensity indirectly indicate a more complete reaction between coal and oxygen. Since the overall change trend of thermogravimetric (TG) curves is similar, further analysis of characteristic parameters is needed to explore the degree of oxidation difficulty of coal samples under different pre-oxidation conditions. The characteristic temperatures of coal can represent the rate or ease of the oxidation process, serving as key parameters for studying the spontaneous combustion behavior of coal. The lower characteristic temperature signifies more intense reaction between coal and oxygen, resulting in a faster progression of the oxidation process. Based on the characteristics of the TG-DTG-DSC curves obtained at a heating rate of 10°C/min and the oxidation properties of coal, the characteristic temperature points during the coal oxidation process are classified. The critical temperature point, denoted as T_1_, refers to the temperature at which the first local maximum mass loss rate occurs on the DTG curve. The activation temperature, denoted as T_2_, corresponds to the temperature of the local minimum mass loss rate. T_3_ represents the ignition temperature. The maximum weight loss temperature, denoted as T_4_, is the temperature at the second local maximum mass loss rate. D_1_ represents the temperature point at which the coal sample attains thermal equilibrium, also known as the initial exothermic onset temperature. D_2_ denotes the minimum point on the differential scanning calorimetric (DSC) curve, corresponding to the temperature at which the maximum heat release intensity occurs.

As can be observed from Table 1, under the coupled influence of pre-oxidation airflow conditions and the maximum temperature attained during the preset oxidation process, the differences between the coal samples and the raw coal at T_1_ and T_3_ were each less than 5°C, whereas T_2_ and T_4_ exhibit relatively larger fluctuations. This indicates that variations in pre-oxidation conditions primarily affected the activation temperature and maximum weight loss temperature of the coal samples. The T_2_ difference between $R_{40}^{200}$ and R_0_ was the largest, with an increase of 8.06°C compared to the raw coal. Compared to other airflow conditions, the $R_{40}^{200}$ coal sample, due to its faster oxidation gas flow rate at 200 mL/min, generated relatively fewer active structures, resulting in a lagged activation temperature point. T_3_ to T_4_ belong to the combustion stage, and the coal sample quality showed an exponential reduction. The T_4_ difference between $R_{110}^{100}$ and R_0_ was the most significant, with a decrease of 21.71°C compared to raw coal. This indicates that, compared to other air flow conditions, the pre-oxidized coal combustion reaction of $R_{110}^{100}$ coal sample under an air flow rate of 100 mL/min is the most intense, resulting in an advancement of the temperature corresponding to the maximum weight loss rate. However, the T_1_, T_2_, T_3_, and T_4_ values of $R_{40}^{25}$, $R_{70}^{25}$, $R_{170}^{25}$, and $R_{110}^{50}$ are all lower than those of R_0_, indicating that the coal samples under the aforementioned four pre-oxidation conditions undergo accelerated oxidation processes compared to raw coal, thus posing a greater risk of re-oxidation.

**Table 1:** Characteristic temperatures under the influence of oxidation conditions.

Air volume (mL/min)	Oxidation temperature (°C)	Characteristic temperature(°C)			
		*T*_1_	*T*_2_	*T*_3_	*T*_4_
raw coal		82.00	197.05	346.99	454.75
25	40	77.95	196.15	345.29	441.10
	70	80.50	195.25	345.05	443.65
	110	83.36	203.51	347.18	443.51
	140	83.83	196.33	345.50	452.08
	170	79.53	195.93	346.65	442.83
50	40	80.44	196.24	348.04	442.84
	70	82.89	196.14	346.52	446.19
	110	79.59	196.13	346.88	444.54
	140	81.33	196.08	347.01	441.78
	170	80.08	203.83	345.64	445.78
100	40	83.57	203.72	344.83	442.07
	70	83.22	196.32	345.16	442.47
	110	83.84	196.19	344.20	433.04
	140	84.25	196.15	345.83	451.90
	170	79.79	202.94	349.19	452.84
200	40	84.06	205.11	344.88	442.86
	70	83.61	203.46	343.93	445.11
	110	81.02	196.07	347.73	433.67
	140	85.24	196.24	342.38	457.09
	170	83.72	196.22	351.18	443.57

The thermal effect parameters of raw coal and pre-oxidized coal samples are presented in Table 2. Compared with raw coal, the initial heat release point of pre-oxidized coal sample shifted to the low temperature area and the maximum heat release intensity was larger, which indicated that pre-oxidized coal samples accumulate heat faster and undergo more complete combustion reactions than raw coal. Based on the analysis of functional group content in coal samples outlined in **Section 3.1**, the pre-oxidized coal samples exhibited lower C = O and sym.-CH_3_ contents compared to the raw coal, while the sym.-CH_2_ content increased. This suggested that the alterations in the aforementioned functional group contents after pretreatment may have influenced the oxidative exothermic process of the pre-oxidized coal samples, resulting in an advancement of the D_1_ temperature point. The D_1_ temperature point of $R_{70}^{25}$ coal sample was the lowest, exhibiting a decrease of 21.90°C compared to the raw coal. This indicated that the coal sample under this pre-oxidation condition underwent a faster rate of thermal accumulation, initiating an exothermic reaction after reaching thermal equilibrium at a lower temperature point. The total heat release of the pre-oxidized coal sample is greater than that of the raw coal, indicating that the pre-oxidized coal sample is more fully combined with oxygen after beginning the exothermic reaction, so as to release more heat.

**Table 2:** Thermal effect parameters under the influence of oxidation conditions.

Air volume (mL/min)	Oxidation temperature (°C)	*D*_1_ (°C)	*D*_2_ (°C)	Maximum exothermic strength (mW/mg)	Heat release (J/g)
raw coal		163.90	465.70	7.39	9001
25	40	146.80	461.65	7.95	9250
	70	142.00	458.95	8.24	9461
	110	144.86	470.21	8.58	10084
	140	147.88	475.18	8.34	10400
	170	145.23	471.63	8.86	10533
50	40	144.34	453.34	8.75	9261
	70	147.84	457.74	8.22	9331
	110	144.84	459.99	8.73	9665
	140	142.68	473.43	8.56	10312
	170	149.08	524.23	8.19	10363
100	40	145.22	463.97	7.89	9573
	70	146.07	466.02	7.59	9292
	110	149.09	457.49	8.58	9557
	140	147.10	477.70	8.15	10481
	170	144.44	464.54	9.46	10560
200	40	142.86	466.26	7.69	9434
	70	145.71	470.16	7.36	9198
	110	144.02	454.67	9.33	9572
	140	148.39	530.29	7.71	10413
	170	148.37	467.42	8.59	10639

The D_2_ temperature point of pre-oxidized coal samples, under a preset temperature condition of 40°C, shifted towards higher temperature regions as the pre-oxidation air flow rate increased beyond 50 mL/min. The peak shape of DSC curve of $R_{40}^{50}$ coal sample was sharper, and the maximum heat release intensity of $R_{40}^{50}$ coal sample was the highest compared with other air volume conditions, and it was increased by 18.40% compared with raw coal. However, under an air flow rate of 25 mL/min, the maximum heat release intensity of $R_{40}^{25}$ coal sample decreased by 9.14% compared to $R_{40}^{50}$ coal sample. Additionally, the D_2_ temperature point of $R_{40}^{25}$ shifted towards the higher temperature region. In this scenario, the excessively low pre-oxidation air flow rate was detrimental to the heat release process of the coal sample. When the preset temperature condition was 70°C, the maximum heat release intensity value of the pre-oxidized coal sample at 25mL/min air volume was the highest, which increases by 11.50% compared with the raw coal, and the D_2_ value was decreased by 6.75°C compared with the raw coal. This indicated that compared to raw coal, the $R_{70}^{25}$ coal sample exhibited a faster reaction rate, achieving the maximum heat release intensity within a shorter period of time, thereby possessing a higher risk of spontaneous combustion. Similarly, at preset temperature conditions of 110°C, 140°C, and 170°C, the maximum heat release intensity values occured at pre-oxidized air flow rates of 200 mL/min, 50 mL/min, and 100 mL/min, respectively. This indicated that the oxidation conditions during the pretreatment process influenced the exothermic intensity of pre-oxidized coal samples, and the optimal airflow conditions varied with different oxidation temperature conditions.

At a pre-oxidation air volume condition of 25 mL/min, the D_2_ value of the pre-oxidized coal sample initially decreased, then increased, and subsequently decreased again with an increase in the preset temperature, with the D_2_ value of $R_{70}^{25}$ coal sample being the smallest. However, compared to coal samples under other preset temperature conditions, $R_{170}^{25}$ coal sample exhibited the highest maximum heat release intensity, which was 19.89% higher than that of the raw coal. This indicated that $R_{70}^{25}$ coal sample attained its maximum heat release intensity within a shorter period, suggesting a faster coal-oxygen reaction rate, whereas $R_{170}^{25}$ demonstrated more stable combustion with a higher heat release intensity. When the pre-oxidized air volume condition was 50mL/min, the D_2_ value of the pre-oxidized coal sample increased with the increase of the preset temperature, and the D_2_ value of the $R_{40}^{50}$ coal sample was the smallest, which was 12.36°C lower than that of the raw coal. Compared to coal samples subjected to other pre-oxidation temperature conditions, $R_{40}^{50}$ exhibited the highest maximum heat release intensity, indicating a faster oxidation rate and a higher risk of spontaneous combustion. Under pre-oxidation airflow conditions of 100 mL/min and 200 mL/min, the D_2_ values of the pre-oxidized coal samples exhibited identical trends with respect to changes in the preset temperature, both achieving their minimum values at a pre-oxidation temperature condition of 110°C. Compared to coal samples under other preset temperature conditions, the maximum heat release intensity of the $R_{170}^{100}$ coal sample was the highest, increased by 28.01% compared to the raw coal. However, under the pre-oxidation airflow condition of 200 mL/min, $R_{110}^{200}$ coal sample exhibited the highest maximum heat release intensity, which was increased by 26.25% compared to the raw coal. This indicated that, under the same pre-oxidation airflow condition, $R_{110}^{200}$ coal sample reacted with oxygen at a faster rate, with a more complete combustion reaction, resulting in a higher risk of spontaneous combustion.

### 3.3 Oxidation kinetics analysis

**Fig 7:**
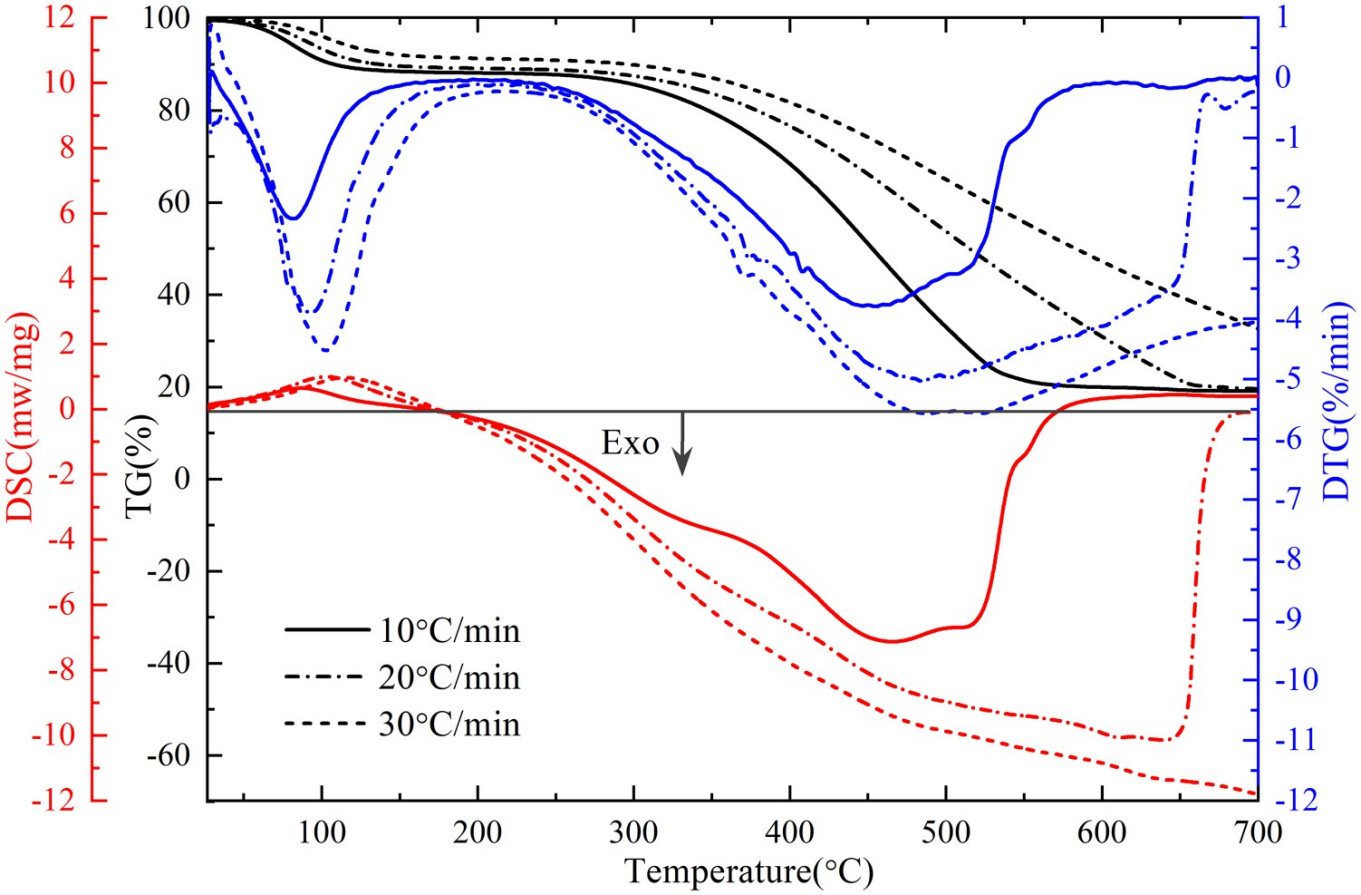
TG-DTG-DSC curves at different heating rates.

The TG-DTG-DSC curves of raw coal under three heating rates (10°C/min, 20°C/min, and 30°C/min) are depicted in Fig 7. As the heating rate increased, both the TG curve and the DTG curve shifted towards higher temperature regions, with the rate of mass loss increasing accordingly. The thermal conductivity of coal itself is poor, and the heat absorption process is relatively short. With the increase of heating rate, the temperature difference between coal and furnace was large, and the reaction between coal and oxygen lagged behind, resulting in obvious lag in TG and DTG curves. The smaller the heating rate, the lower the peak value of the DSC curve and the more intact the peak shape. As the heating rate increased, the energy transferred from the external heat source to the coal body rised, resulting in an elevation of the instantaneous heat flow rate of the coal.

**Fig 8:**
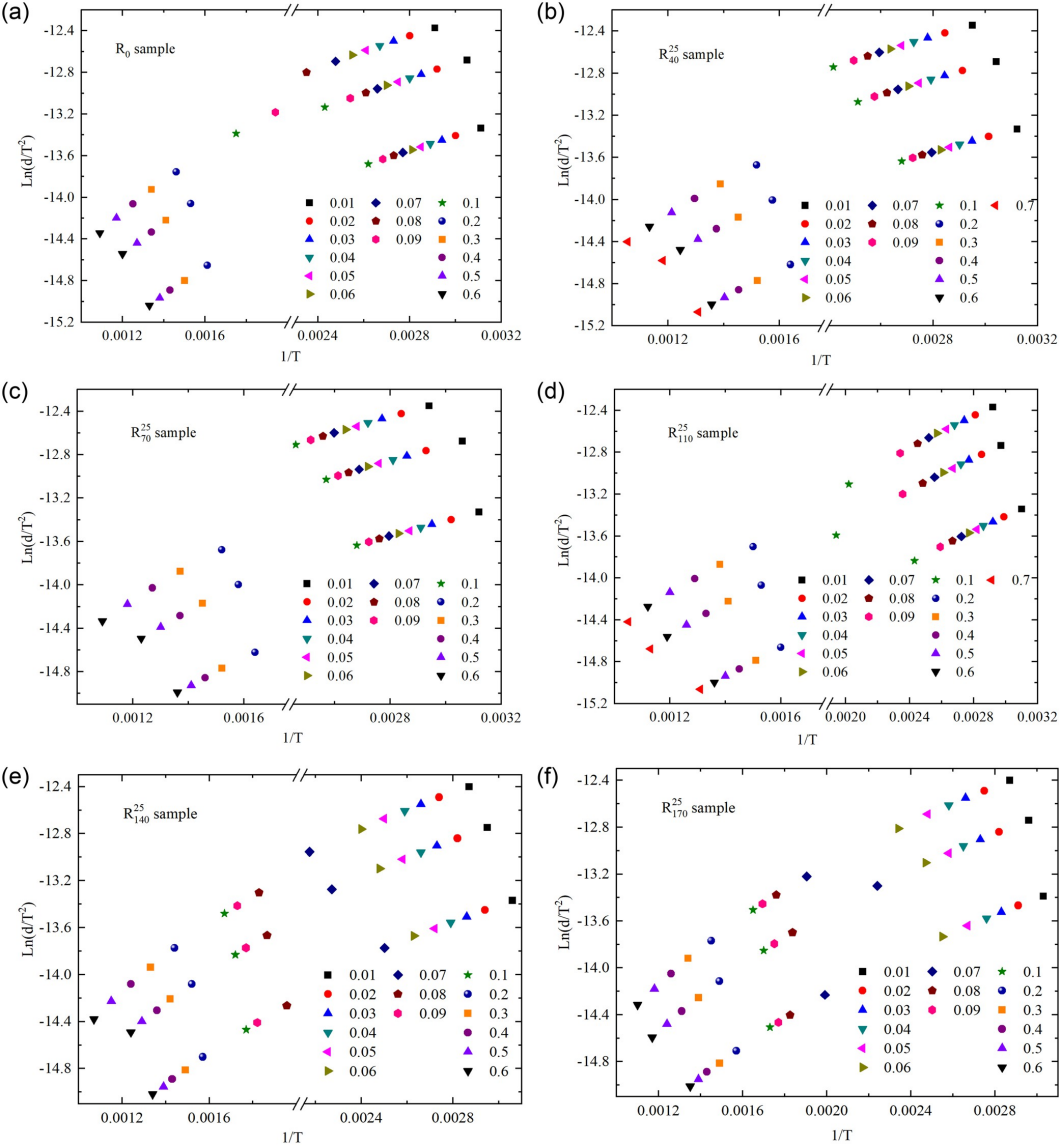
Fitting process of activation energy of samples at different conversion rates.

Linear fitting was performed on the TG curve data corresponding to three heating rates at the same conversion rate. The fitting processes of pre-oxidized coal samples under a pre-oxidation air flow rate of 25 mL/min and raw coal are exemplified, as shown in Fig 8. The linear relationship exhibited good performance at conversion rates of 0.01–0.06 and 0.2–0.6, and not all pre-oxidized coal samples can achieve a conversion rate of 0.7 prior to the final experimental temperature. The termination temperature range for pre-oxidation was 40°C to 170°C, with a maximum attainable conversion rate of 0.06 for the pre-oxidized coal samples prior to reaching 170°C. Based on the analysis of characteristic temperatures presented in **Section 3.2**, it is observed that coal samples undergo low-temperature oxidation reactions within a temperature range of approximately 46.89°C to 169.64°C when the conversion rate is between 0.01 and 0.06. Similarly, when the conversion rate is within the range of 0.2 to 0.6, the coal samples undergo high-temperature oxidation reactions within a temperature range of approximately 346.36°C to 689.84°C. Therefore, with a step size of 0.01 for the conversion rate, analyzing the average activation energy within the range of 0.01–0.06 can approximately reflect the changes in activation energy of pre-oxidized coal samples during the low-temperature oxidation stage. Similarly, with a step size of 0.1 for the conversion rate, analyzing the average activation energy within the range of 0.2–0.6 can approximately indicate the variations in activation energy of pre-oxidized coal samples during the high-temperature oxidation stage.

**Fig 9:**
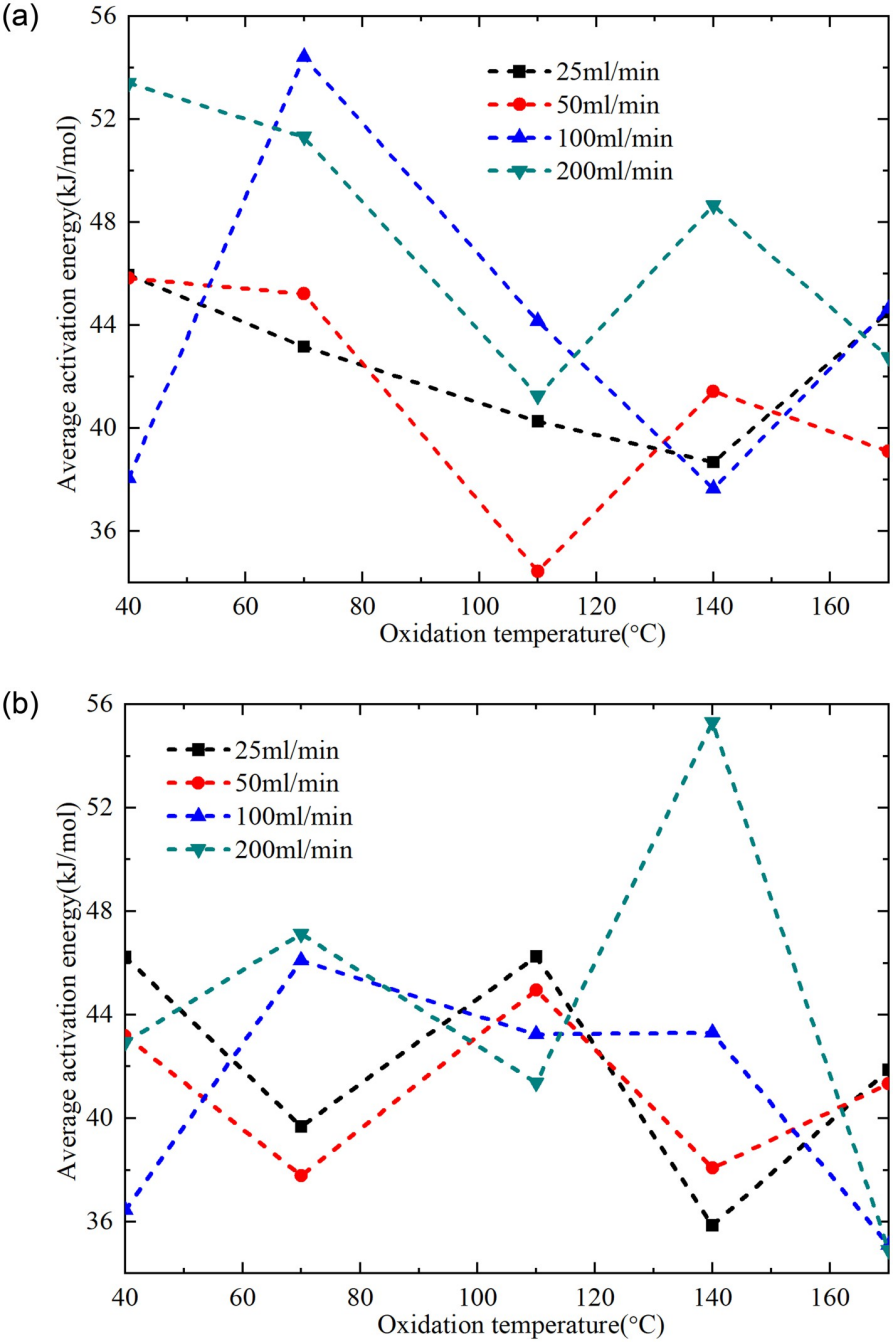
Change of average activation energy with oxidation conditions.

Based on KAS method, the apparent activation energy can be calculated from the slope of the fitted curve and the average activation energy of different conversion ranges can be further obtained. The average activation energy of raw coal within the conversion rate range of 0.01–0.06 was 34.50 kJ/mol, while the conversion rate range of 0.2–0.6 was 37.59 kJ/mol. The variations in average activation energy of pre-oxidized coal samples under different oxidation conditions are illustrated in Fig 9. As can be seen from Fig 9a, the average activation energy of pre-oxidized coal samples under 25mL/min and 100mL/min air volume conditions at the low temperature oxidation stage was the lowest when the preset temperature was 140°C, and increased by 4.17kJ/mol and 3.13kJ/mol compared with raw coal, respectively. The average activation energy of pretreated coal samples under 50mL/min and 200mL/min air volume conditions was the lowest when the preset temperature is 110°C, and increased by -0.06kJ/mol and 6.74kJ/mol compared with raw coal, respectively. Except for the slightly lower average activation energy of the coal sample oxidized to 110°C under an air flow rate of 50 mL/min compared to raw coal, the average activation energy of pre-oxidized coal samples during the low-temperature oxidation stage was generally higher than that of raw coal. Compared to raw coal, the coal sample oxidized to 110°C under an air flow rate of 50 mL/min exhibited an increase in the content of OH…OH, enhanced oxidation reactivity, advanced critical temperature and active temperature, resulting in a reduction in the energy required for the low-temperature oxidation stage. Compared with the coal samples under other pre-oxidation conditions, the average activation energy of the coal sample oxidized to 70°C under 100mL/min air volume was the highest at the low temperature oxidation stage, which may be mainly related to the content proportion of Ar-C-O-, bsym.-CH_3_, -CH_2_&-CH_3_, C = O and Free-OH in the coal sample.

The average activation energy of the pre-oxidized coal samples at the conversion rate of 0.2 to 0.6 is depicted in Fig 9b. Under 25mL/min and 50mL/min air volume conditions, the average activation energy of pre-oxidized coal samples in the high temperature oxidation stage showed a trend of first decreasing and then increasing with the increase of preset temperature conditions in the range of 40°C-110°C and 110°C-170°C. It was opposite to 100mL/min and 200mL/min air volume conditions. In the high temperature oxidation stage, the combustion reaction of coal is mainly involved, so the diffusion and binding of oxygen to the pores of coal are affected by the air volume. At a preset termination oxidation temperature of 110°C, the difference in average activation energy among pre-oxidized coal samples during the high-temperature oxidation stage under four airflow conditions was minimal, which may be related to structural changes in the coal samples at 110°C.

## 4. Conclusions

1. Variations in air volume conditions primarily influence the content of Ar-C-O- and -CH_2_&-CH_3_ in the oxygen-containing functional group region. Except for the coal sample oxidized to 110°C under a 50 mL/min airflow condition, where the content of OH…OH group increased by 0.04 times compared to the raw coal, the content of component OH…OH group in coal samples under other pre-oxidation conditions decreased by 1.68% to 57.73% relative to the raw coal.
2. Changes in pre-oxidation conditions primarily influence the activation temperature and maximum weight loss temperature of coal samples. The differences in critical temperature and ignition temperature between pre-oxidized coal samples and raw coal are both less than 5°C. Coal samples oxidized to 40°C, 70°C and 170°C at 25mL/min air volume, and coal samples oxidized to 110°C at 50mL/min air volume, respectively, have lower critical temperature, active temperature, ignition point and maximum weight loss temperature than raw coal, making them more prone to oxidative spontaneous combustion.
3. Except for the coal sample oxidized to 110°C under an air volume of 50 mL/min condition, which exhibited an average activation energy 0.06 kJ/mol lower than that of raw coal, the pre-oxidized coal samples displayed average activation energies greater than raw coal within the conversion rate range of 0.01–0.06. The average activation energy of the pre-oxidized coal sample under the 25mL/min and 50mL/min air volume conditions shows the same trend with the increase of the pre-oxidizing temperature when the conversion rate is 0.2–0.6, but the change trend is opposite to that under the 100mL/min and 200mL/min air volume conditions.

## Supporting information

S1 File Thermogravimetric data of coal samples at different heating rates. (ZIP)
